# Supplementary material for: A set of multi-entry identification keys to African frugivorous flies (Diptera, Tephritidae)
Source: Zookeys. 2014 Jul 24;(428):97–108. doi: 10.3897/zookeys.428.7366 (PMC4143993; doi:10.3897/zookeys.428.7366)
Supplement: Supplementary material 6 — Key to Ceratitis [file zookeys-428-097-s006.zip › SF6_ZooKeys_key to Ceratitis/key/SF6_ZooKeys_key to Ceratitis/Media/Html/Ceratitis melanopus .htm]

Ceratitis (Ceratalaspis) melanopus (Hering)


***Ceratitis*** **(*Pterandrus*) *melanopus*
(Hering)**

*Pardalaspis melanopus* Hering, 1942: 282.

Body
length: male 3.35, female 5.40 mm; wing length: male 4.20, female 5.60 mm.

Male

Head: Antenna brownish. First flagellomere twice as long as
pedicel. Arista with short rays. Frons convex; yellowish orange with weak
silvery shine; with short scattered setulae of same color as frons. Frontal
setae well developed. Face yellowish orange.

Thorax:
Postpronotal lobe white, not spotted. Scutal pattern, ground color darkish gray
to brown, microtrichose areas with ashgray shine, poorly developed lateral
yellow lines, prescutellar white markings separate, with grayish area in
between. Scapular setae dark. Scutellum white, basally without dark spots,
apically more yellowish with three separate spots.

Legs:
orange to brown; setation typical for subgenus, dark. Without feathering.

Wing:
Bands brown. Interruption between marginal and discal bands near vein R_1_
clear and complete; cubital band free; medial band absent; crossvein R-M proximal
to middle of discal cell. Apex of vein R_1_ distal to level of
crossvein R-M. Crossvein DM-Cu oblique posterobasally.

Abdomen:
Mostly pale orange. Tergite 3 with posterior half brown. Setation and banding
typical for subgenus. Male terminalia not dissected.

Female

As
male except for the following characters: Rays on arista longer but not
plumose. First flagellomere longer. Face yellow. Scutal pattern slightly
different, with more obscured orange lateral stripes, area between prescutellar
markings more orange. Marginal band partly interrupted. Oviscape about as long
as preabdomen. Aculeus at least eight times longer than wide; tip with distinct
apical indentation and lateral margin more or less straight.

(Description
after De Meyer & Freidberg, 2006)
